# Innovative Elastomers with Antimicrobial Activity May Decrease Infection Risks during Milking

**DOI:** 10.3390/pathogens12121431

**Published:** 2023-12-08

**Authors:** Gabriele Meroni, Valerio Sora, Francesca Zaghen, Giulia Laterza, Piera Anna Martino, Alfonso Zecconi

**Affiliations:** 1 Department of Biomedical, Surgical and Dental Sciences-One Health Unit, School of Medicine, University of Milan, Via Pascal 36, 20133 Milan, Italy; gabriele.meroni@unimi.it (G.M.); valerio.sora@unimi.it (V.S.); francesca.zaghen@unimi.it (F.Z.); giulia.laterza@unimi.it (G.L.); piera.martino@unimi.it (P.A.M.); 2 Department of Clinical and Community Sciences, School of Medicine, University of Milan, Via Celoria 22, 20133 Milan, Italy

**Keywords:** One Health, mastitis, antimicrobial resistance, elastomers, milking, liners

## Abstract

Contagious pathogens are very costly to dairy herds, and they may have zoonotic and reverse-zoonotic potentials and may contribute to the spread of antimicrobial resistance. One of the most important risk factors for spreading these infections is milking, when liner contamination may transfer the pathogens from infected to healthy cows. There is no effective protocol to prevent the transmission of infection without the segregation of infected cows. Recently, the availability of elastomers with patented antimicrobial components in their formulations has allowed the exploration of alternative methods to reduce the risk of infection. Two different types of elastomers (rubber and silicone) and nine different formulations were challenged with three major mastitis pathogens (*S. aureus*, *S. agalactiae*, and *E. coli*). The results that were obtained in this study were interesting and unexpected. Indeed, to our knowledge, this is the first study to show that basic rubber materials have intrinsic antimicrobial activity. Silicone elastomers did not exhibit the same levels of bactericidal activity, although they did exhibit some antibacterial capacity. A significant decrease in bacterial survival curves was observed for all the formulations tested when antimicrobial components were added. The different results observed for the various products are likely due to the different formulations and diverse manufacturing processes. The availability of these new materials that significantly reduce the bacterial load on the liner surface may reduce the risk of spreading intramammary infections during milking. This would be an important step forward in achieving global sustainability of dairy herds, consistent with the objectives of One Health, by reducing the risks of zoonotic diseases and antimicrobial treatments.

## 1. Introduction

Mastitis remains one of the most important economic burdens for dairy farmers [1,2,3]. Among all the bacteria involved in mastitis epidemiology, contagious pathogens (*S. aureus* and *S. agalactiae*) represent an important group because, in addition to the high direct and indirect costs of the mastitis they induce in dairy herds, they may also have zoonotic and reverse-zoonotic potentials and may contribute to the spread of antimicrobial resistance (AMR), as shown by several studies [4,5,6,7]. From a One Health perspective, controlling and eradicating these infections has several benefits, such as reducing antimicrobial use and resistance, improving milk quality and yield, increasing the nutritional value of milk, and reducing the risk of human infections [6,8,9].

One of the most important risk factors for the spread of these infections is milking, especially liner contamination, which can transfer pathogens from infected to healthy cows [6,10,11,12]. The segregation of infected cows was shown to be an effective way to decrease the spread of infection [6,13]. However, segregation requires at least three groups of cows (healthy, infected, and to be diagnosed) and an increase in labor for herdsmen. The use of backflush systems, which clean and disinfect milking units between cows, has been suggested as a way to reduce the risk of infection without segregation. Unfortunately, the results of this practice are mixed, and these systems alone do not reduce the incidence of intramammary infections [14,15,16]. These risks are even higher when automated milking systems are used, as a single milking unit can milk up to 60–70 cows, which increases the risk of infection and the incidence of chronic mastitis [17,18,19].

Rubber and silicone elastomers are largely present in milking machines (either conventional or automated) in the form of short and long milk tubes, gaskets, and liners. All these components are subject to contamination by bacteria present in the milk (commensals or pathogens). Therefore, the milking machines must be cleaned and disinfected regularly to reduce the number of bacteria in the milk. These procedures affect the elastomer surface, increasing its receptiveness to bacterial contamination, and they do not guarantee the absence of pathogens on the elastomer surface [20,21]. Recently, the production of elastomers containing antimicrobial components in their formulation has increased the interest in the application of these materials in medical devices and food production [22,23,24]. Among these new products, an innovative patented technology (Scudo Technologies, Adrara San Martino, Italy) has allowed the development of elastomers with antimicrobial capability that can be used to produce both rubber and silicone liners.

The availability of these materials allowed us to test them in vitro on three major mastitis pathogens (*S. aureus*, *S. agalactiae*, and *E. coli*) as a first step in identifying the elastomers with the highest antibacterial activity. This preliminary study was critical in designing field studies to evaluate their ability to reduce the risk of infection during milking.

## 2. Materials and Methods

### 2.1. Elastomer Characteristics

Two different types of elastomers were considered: rubber and silicone. Two different rubber elastomers were developed, while seven different silicone formulations were developed. In each formulation, a different patented antimicrobial additive (PAA) was added.

A detailed description of all formulations and relative PAAs is reported in Table 1 and Table 2.

All the elastomers were produced with ingredients approved by the Food and Drug Administration for use in food production. The presence of silver components in the different formulations does not represent a health risk as hypothesized for other devices [25] because these components are not added as silver nitrate or nanoparticles that may represent a risk to the aquatic environment. In addition, the elastomers have been tested for specific migration according to the UNI EN 13130-1:2005 procedure, with values <0.0001 mg/kg. The potential environmental risks are managed through the proper disposal of rubber and silicone devices, which follows specific regulations, at least in Europe.

### 2.2. Bacterial Strains and Culture Conditions

The potential antimicrobial activity of elastomers was tested on *Staphylococcus aureus* ATCC 6538, *Escherichia coli* ATCC 25922, and *Streptococcus agalactiae* ATCC 13813, which represent major mastitis pathogens. Bacteria were stored at −20 °C in 25% (*v*/*v*) glycerol, thawed at room temperature, and 10 µL were plated on tryptic Soy Agar (Microbiol, Cagliari, Italy) with aerobic incubation at 37 °C overnight. After incubation, the bacterial inoculum was prepared by suspending colonies in sterile saline solution (NaCl, 0.9% *w*/*v*) to reach a concentration of 0.5 McFarland units (equivalent to 1.5 × 10^8^ CFU/mL) determined with a densitometer (BioSan, Medical-Biological Research and Technologies, Riga, Lettonia). Serial dilutions were performed to obtain final concentrations of 10^4^ and 10^3^ CFU/mL. These concentrations were selected to be as close as possible to the values observed under field conditions.

### 2.3. Preparation of Silicone Sheets and Microplate Loading

Squares of 1.21 cm^2^ from each elastomer sheet were cut with sterile scissors and positioned at the bottom of the wells of 24-well plates (Cellstar, Greiner bio-one, Milan, Italy). Then, 1 mL of each bacterial suspension was dispensed over each square. For each 24-well plate, one sterility control was included. Four different time points were used to assess the antibacterial activity of the silicone sheets: T0 (contact), T1 (1 h after contact), T2 (6 h after contact), and T3 (24 h after contact). At each time point, 50 µL of bacterial suspension was collected from the wells, diluted tenfold in microtubes, plated on tryptic Soy Agar (Microbiol, Cagliari, Italy), and aerobically incubated at 37 °C for 24 h. After incubation, the colonies on each plate were counted, and the final results were expressed in CFU/mL. Each elastomer and pathogen were tested in triplicate for each time point.

### 2.4. Statistical Analysis

Data were collected in a database, and the killing rate was calculated using the following formula:

(1) $$K\left(\%\right)=\left(1-\frac{{\mathrm{CFU}}_{\mathrm{tn}}-{\mathrm{CFU}}_{t0}}{{\mathrm{CFU}}_{t0}}\right)\times 100$$

where K(%) is the reduction in the bacterial population (percentage, %), CFU_tn_ is the bacteria concentration (CFU/mL) at time n > 0, and CFU_t0_ is the initial bacteria concentration (CFU/mL).

The Kaplan–Meier method was applied to compare the killing activity during the entire follow-up period (24 h) (XLstat 2023.1.4 Addinsoft, New York, NY, USA). The Kaplan–Meier analysis allows the comparison of populations through their survival curves, even in the case of irregular detection time. 

The mean killing rate (K%) represents the end point evaluation of the antimicrobial activity of the elastomers, whereas the Kaplan–Meier medians represent the trend of activity through time. 

## 3. Results

In this study, nine different elastomers (two rubber and seven silicone formulations) were challenged with three major bovine mastitis pathogens. The results of the antibacterial activities, evaluated in triplicate and compared to a control, are reported in the following figures and tables based on the different formulations of the elastomers as described in Table 1 and Table 2.

### 3.1. Rubber Elastomers

**Figure 1:**
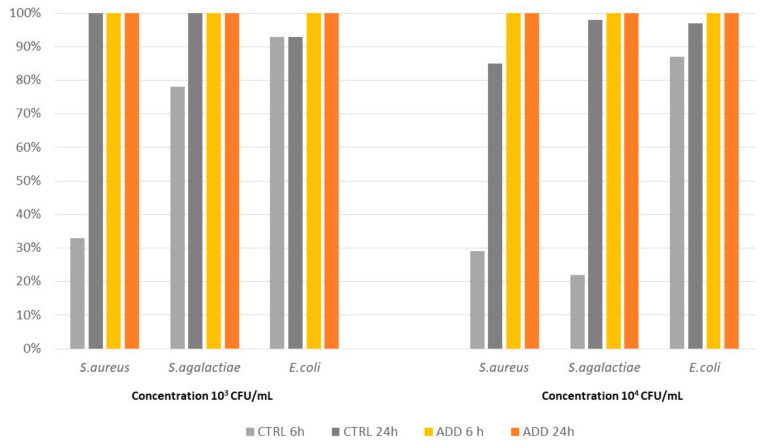
Killing activity of elastomer R1 and its control on *S. aureus*, *S. agalactiae*, and *E. coli* at two different initial concentrations (10^3^ CFU/mL and 10^4^ CFU/mL), measured at two different time points after contact (6 h and 24 h).

The results of the challenge of rubber elastomer R1 without PAA with the three pathogens considered showed unexpected results. Indeed, antimicrobial activity was observed in the control with a killing rate of up to 100% for *S. aureus* and *S. agalactiae* at 24 h post contact (pc), when the initial concentration was 10^3^ CFU/mL. A relatively high killing rate (range 85–98%) was also observed when the 10^4^ CFU/mL concentration was used. As expected, the addition of antimicrobial components increased the killing rate to 100% at both 6 h and 24 h pc for all three pathogens (Figure 1).

Table 3 reports the result of the Kaplan–Meier survival curve analysis. The controls confirmed the presence of antibacterial activity against the three pathogens considered but at a significantly lower level compared to the PAA-added rubber, which killed all bacteria within 24 h pc. It should also be noted that the curve analysis estimated a killing activity of 50% at 6 h pc for all the pathogens considered.

**Figure 2:**
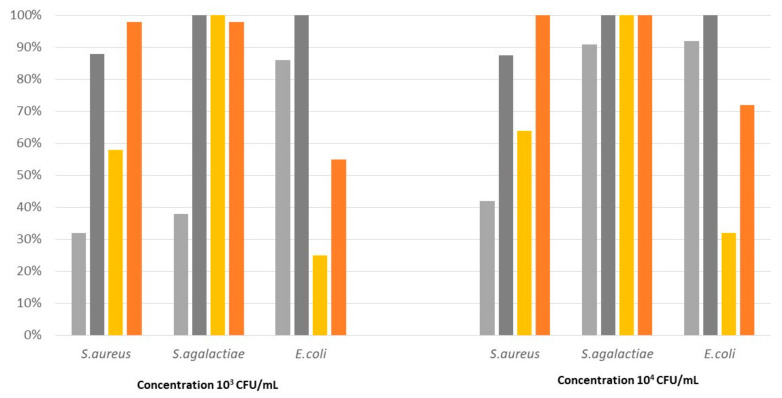
Killing activity of elastomer R2 and its control on *S. aureus*, *S. agalactiae*, and *E. coli* at two different initial concentrations (10^3^ CFU/mL and 10^4^ CFU/mL), measured at two different time points after contact (6 h and 24 h).

The second rubber elastomer (R2) showed a different antimicrobial activity than the R1 elastomer. In fact, the killing activity of the control was less than 90% at 24 h pc for *S. aureus*, whereas the PAA-added elastomer showed an activity close to or equal to 100% only for *S. agalactiae* (Figure 2), which was significantly higher than the control (Table 4). Finally, the activity against *E. coli* was significantly lower for the PAA-added product compared to its control (Table 4 and Figure 2).

### 3.2. Silicone Elastomers

**Figure 3:**
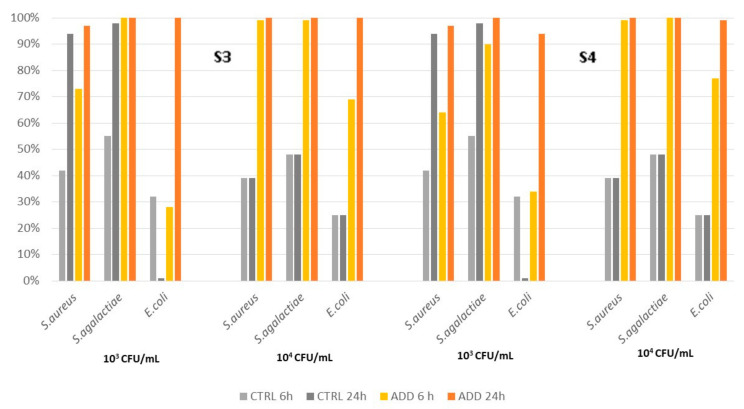
Killing activity of elastomers S3 and S4 and its control on *S. aureus*, *S. agalactiae*, and *E. coli* at two different initial concentrations (10^3^ CFU/mL and 10^4^ CFU/mL), measured at two different time points after contact (6 h and 24 h).

The evaluation of the antimicrobial activity of the silicone elastomers S3 and S4 showed greater differences compared to the rubber elastomers. Indeed, the control product showed low antimicrobial activity against the three pathogens considered, which was in the range of 1–98% for a bacterial concentration of 10^3^ CFU/mL and 29–48% for the higher concentration. An activity > 90% was observed only against *S. aureus* (Figure 3). PAA-added silicone S3 showed a significantly higher antimicrobial activity against all three pathogens compared to the control (*p* < 0.0001) at 24 h pc (Table 5). Silicone S4 had a slightly lower killing activity when compared to S3, showing efficacy against *S. agalactiae* at 6 h pc, but very few colonies were present at 24 h pc when challenged with *E. coli*. Again, the differences between the PAA-added and control silicones were statistically different according to the Kaplan–Meier analysis (*p* < 0.0001).

**Figure 4:**
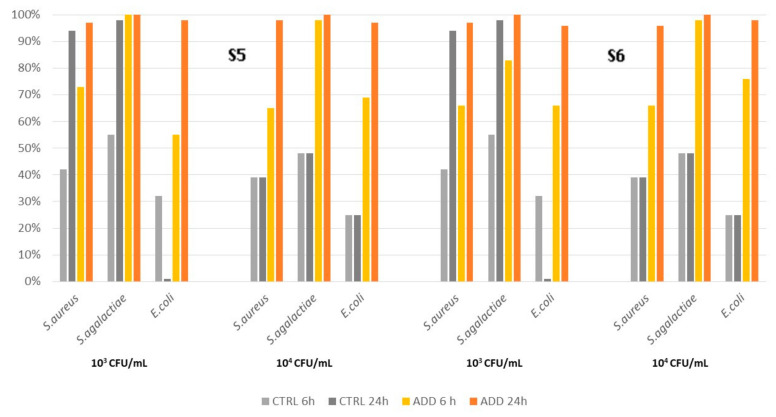
Killing activity of elastomers S5 and S6 and its control on *S. aureus*, *S. agalactiae,* and *E. coli* at two different initial concentrations (10^3^ CFU/mL and 10^4^ CFU/mL), measured at two different time points after contact (6 h and 24 h).

The antimicrobial activity of PAA-added silicones S5 and S6 (Figure 4) was slightly lower than that observed for S3 and S4, reaching a level of 100% against *S. agalactiae* only at 24 h pc. Despite the different formulations of the two silicones, their antimicrobial activity was similar (Table 6), although S6 had a faster action with lower survival rates at 6 h pc compared to S5. The survival curves of all additive silicones were significantly different from the control according to the Kaplan–Meier analysis (*p* < 0.0001).

Silicones have a very low basal antibacterial activity, and the additives play a major role in modifying it. In fact, silicones without additives could not kill all the bacteria at 24 h pc, as observed in some cases for the rubber ones. It should be noted that the basal formulation was ineffective against *E. coli*, but some activity was observed for the other two pathogens. Different results were obtained by adding PAA to the base silicone formulation. The addition of silver phosphate glass, silver chloride, and zinc pyrithione significantly decreased survival rates (S3). However, when magnesium oxide and zinc oxide were added to the formulation (S4), very little difference was observed. The same formulation as S4 but without zinc oxide (S5) had similar results as S4, as did the same formulation with zinc oxide instead of zinc pyrithione (S6).

**Figure 5:**
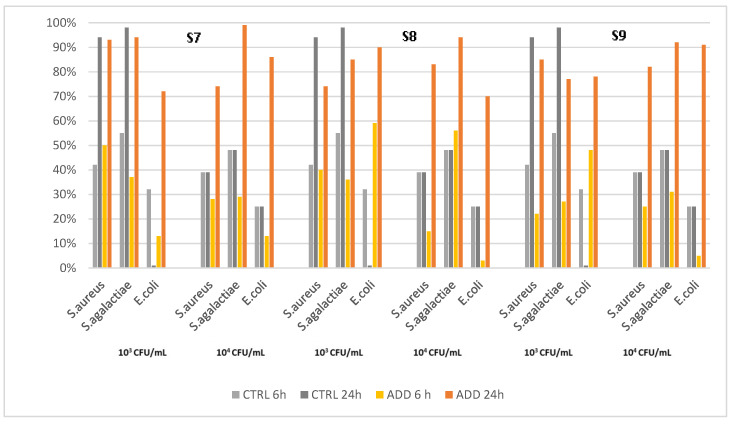
Killing activity of elastomers S7–S9 and their controls on *S. aureus*, *S. agalactiae*, and *E. coli* at two different initial concentrations (10^3^ CFU/mL and 10^4^ CFU/mL), measured at two different time points after contact (6 h and 24 h).

The fourth set of analyses concerns a silicone elastomer formulated with three different concentrations of the same PAA. The evaluation results showed different patterns of killing activity (Figure 5), and in very few cases, the killing activity reached the 100% level, although a significant difference from the control was always observed through the Kaplan–Meier analysis (Table 7). The PAA-added silicones were more active against *E. coli* than the control, regardless of additive concentration, but there was no correlation between concentration and killing rate. Only S7 showed a high kill rate against *S. agalactiae*, and the control showed greater activity against *S. aureus* than all three PAA-added silicones.

## 4. Discussion

The alarming worldwide spread of AMR demands the creation of novel protocols and tools aimed at preventing and controlling microorganisms and thus decreasing the need for antimicrobials. One promising avenue towards achieving this goal is the development of elastomers with antimicrobial properties [24,26,27]. 

These materials have wide potential applications, including in the food industry and milk production. Contagious pathogens still exhibit a high prevalence in many herds [6,8,28,29], and the two major contagious pathogens (*S. aureus* and *S. agalactiae*) may have a zoonotic potential both directly and as vectors of AMR [4,5,7,30].

Milking is a critical stage for spreading infectious pathogens within the dairy herd. Segregating cows during milking is the only effective way to significantly decrease this risk [6,13]. However, this method is often seen as troublesome in many dairy herds and requires additional labor. Other methods, such as cluster disinfection, have produced controversial results [14,15,16]. The risk of transmission increases when automated milking systems are used because proper segregation is practically impossible to achieve, owing to the requirement of maintaining a consistent number of cows per unit, and since hygiene procedures are not always effective [31,32,33]. 

The availability of elastomers possessing antimicrobial properties applicable to rubber and silicone components of miking machines could potentially reduce the risk of infection transmission during milking, ultimately decreasing the need for additional control measures like segregation. The opportunity to compare the killing capacity of several rubber and silicone elastomers with different antibacterial additives led to unexpected results. To the best of our knowledge, this is the first study to show that basic rubber materials possess an inherent antimicrobial activity that, in some cases, can be as high as 100%. In contrast, silicone elastomers without PAA displayed lower bactericidal properties. The intrinsic antimicrobial activity may justify the high level of activity seen for both rubber elastomers tested when antimicrobial components were added. The inclusion of PAA resulted in a markedly increased rate of lethality for both elastomers. R1 exhibited a higher degree of killing efficacy compared to R2, even though both possess the same concentration of PAA, owing to the differing basal compositions. Bacteria, as expected, also play a role. Indeed, while R1 led to similar survival curves for all three pathogens, R2 showed the highest killing activity for *S. agalactiae* and the lowest activity for *E. coli*.

Finally, the addition of different concentrations of silver phosphate glass together with zinc pyrithione (S7–S9) but without the other antimicrobial components, did not result in the expected proportional decrease in survival rates. Interestingly, the elastomers with lower concentrations of additives exhibited greater activity than those with higher concentrations. In addition, the inclusion of just two constituents (silver phosphate glass and zinc pyrithione) yielded a decrease in bactericidal activity compared to the other compositions.

The different results may also be due to the diverse chemical reactions that take place during manufacturing and may affect the antimicrobial efficacy of the single and combined components, as previously observed [23,34,35].

The comparison of the formulations enables us to identify at least one rubber elastomer with the highest in vitro antimicrobial activity, represented by the R1 formulation that includes zinc, silver, and magnesium components as PAA. When considering silicone elastomers, identifying the best formulation is not as straightforward. Indeed, both S4 and S5 elastomers exhibited similar antimicrobial activity. This result was expected given that the only difference between the two was the presence of zinc oxide in S4 and not in S5. When determining the optimal product, other factors such as cost need to be considered, although this information was not provided by the producer.

The development of new antibacterial elastomers should involve a precise evaluation of at least the in vitro effects of the component mixture and manufacturing process on antimicrobial capability. Thus, a comprehensive assessment of various components and their interplay is necessary to produce effective antibacterial elastomers. Achieving an optimal component combination is crucial for antimicrobial activity and is not solely reliant on the additive effects.

This study is limited to being an in vitro study covering only three pathogens, although it may be representative of other mastitis pathogens (e.g., coagulase-negative Staphylococci, environmental Streptococci, and coliforms). One key factor absent in comparison to field studies is the cleaning process, which could have an impact on elastomer conditions and contribute to reducing bacterial loads on its surface. Given the limitations of this study, conducting an in vitro assessment is crucial to determine the most effective formulation before conducting field trials, which should also take into account potential confounding factors.

## 5. Conclusions

Controlling infectious diseases with zoonotic and antimicrobial resistance potential in dairy herds is critical, and it is a major requirement in a One Health approach. This approach also implies that the dairy industry must ensure the supply of healthy and safe products, free from pathogens and residues while maintaining a high nutritional value. The availability of new tools, such as elastomers with antimicrobial properties that can be used to manufacture liners, is a progression towards these targets. The potential decrease in the bacterial load on the surface of these liners may reduce the risk of infection spread without compromising milk safety and quality.

## 6. Patents

International application: PCT/IB2023/050905 “A composition suitable for the production of a thermosetting elastomer with antimicrobial capabilities by means of vulcanization by molding”.

International patent classification: C08K3/04 C08K3/32 C08K5/00 C08L7/00 C08L23/16 C08L27/16 C08L43/04.

## Figures and Tables

**Table 1:** Description of formulations for the 2 different rubber elastomers considered. The formulation without additives represented the control.

Main Components	Patented Antimicrobial Additive (1%)	Acronym (in Tables and Figures)
NBR Acrylonitrile Copolymer (CAS 9003-18-3) 50–60% Carbon black (CAS: 1333-86-4) 3–7% Plasticizer (CAS 103-23-1) 6–10% Calcined kaolin (CAS 92704-41-1) 20–24% Precipitated silica (112926-00-8) 4–6% Stearic acid (CAS: 57-11-4) 0.1–1% TMTM (CAS 97-74-5) 0.1–1% ZDBC (CAS 136-23-2) 0.1–1% Sulfur (CAS 7704-14-9) 0.1–2%	Zinc oxide (CAS 1314-13-2) Magnesium oxide (CAS 1309-48-4) Silver chloride (CAS 7783-90-6) Silver phosphate glass (CAS- 308069-39-8) Zinc pyrithione (CAS 13463-41-7)	R1
Butilic rubber (CAS 9010-85-5) 54–57% Carbon black (CAS: 1333-86-4) 26–29% Calcium carbonate (CAS 1317-65-3) 9.5–11% Stearic acid (CAS: 57-11-4) 0.1–1% ZOEC (CAS 14324-55-1) 0.25–1% TMDM (CAS 137-26-8) 0.4–1.8% Sulfur (CAS 7704-14-9) 0.1–2%	Zinc oxide (CAS 1314-13-2) Magnesium oxide (CAS 1309-48-4) Silver chloride (CAS 7783-90-6) Silver phosphate glass (CAS 308069-39-8) Zinc pyrithione (CAS 13463-41-7)	R2

**Table 2:** Description of the formulations for the 7 different silicones considered. The formulation without additives represented the control.

Main Components	Patented Antimicrobial Additive	Acronym (in Table and Figures)
Silicone rubber (CAS: 63394-02-5) 95–99% 50% Dicumyl peroxide (CAS 80-43-3)– Organic peroxides, H242 50%2.5-Dimethyl-2,5-di(tert-butylperox) hexane (CAS 78-63-7)–Organic peroxides, H24	Silver chloride (CAS 7783-90-6) Silver phosphate glass (CAS 308069-39-8) Zinc pyrithione (CAS 13463-41-7)	S3
	Zinc oxide (CAS 1314-13-2) Magnesium oxide (CAS 1309-48-4) Silver chloride (CAS 7783-90-6) Silver phosphate glass (CAS 308069-39-8) Zinc pyrithione (CAS 13463-41-7)	S4
	Magnesium oxide (CAS 1309-48-4) Silver chloride (CAS 7783-90-6) Silver phosphate glass (CAS 308069-39-8) Zinc pyrithione (CAS 13463-41-7)	S5
	Zinc oxide (CAS 1314-13-2) Magnesium oxide (CAS 1309-48-4) Silver chloride (CAS 7783-90-6) Silver phosphate glass (CAS 308069-39-8)	S6
	2% Silver phosphate glass (CAS 308069-39-8) Zinc pyrithione (CAS 13463-41-7)	S7
	Same as above but at 3% concentration	S8
	Same as above but at 4% concentration	S9

**Table 3:** Comparison of median survival rates of the three major mastitis pathogens in response to the additivated R1 elastomer vs. control calculated using the Kaplan–Meier method.

Pathogen	Median Survival Rate (%)						Control vs. R1 (PAA-Added) *p* =
	Control			R1			
*S. aureus*	T1 ^a^	T6	T24	T1	T6	T24	
10^3^ CFU/mL	82 ^b^	81	0	70	50	0	<0.0001
10^4^ CFU/mL	95	75	14	78	50	0	<0.0001
*S. agalactiae*							
10^3^ CFU/mL	96	61	0	70	50	0	<0.0001
10^4^ CFU/mL	97	86	2	67	50	0	<0.0001
*E. coli*							
10^3^ CFU/mL	79	53	7	67	50	0	<0.0001
10^4^ CFU/mL	80	56	3	67	50	0	<0.0001

^a^ Time post-contact. ^b^ Standard error of the survival rate is in the range of 0–0.05% for all the values.

**Table 4:** Comparison of median survival rates of the three major mastitis pathogens in response to the additivated R2 elastomer vs. control calculated using the Kaplan–Meier method.

Pathogen	Median Survival Rate (%)						Control vs. R2 (PAA-Added) *p* =
	Control			R2			
*S. aureus*	T1 ^a^	T6	T24	T1	T6	T24	
10^3^ CFU/mL	100 ^b^	84	12	95	71	2	<0.0001
10^4^ CFU/mL	92	78	12	86	65	0	<0.0001
*S. agalactiae*							
10^3^ CFU/mL	79	63	0	75	0	0	<0.0001
10^4^ CFU/mL	85	55	0	73	0	0	<0.0001
*E. coli*							
10^3^ CFU/mL	88	57	0	90	85	44	<0.0001
10^4^ CFU/mL	88	54	0	94	82	28	<0.0001

^a^ Time post-contact. ^b^ Standard error of the survival rate is in the range of 0–0.05% for all the values.

**Table 5:** Comparison of median survival rates of the three major mastitis pathogens in response to the additivated S3 and S4 elastomers and control calculated using the Kaplan–Meier method (differences between PAA-added and control elastomers were all significant at *p* = 0.001).

Pathogen	Median Survival Rate (%)								
	Control			S3			S4		
*S. aureus*	T1 ^a^	T6	T24	T1	T6	T24	T1	T6	T24
10^3^ CFU/mL	98 ^b^	79	6	87	62	3	95	68	3
10^4^ CFU/mL	86	77	8	80	40	0	78	51	0
*S. agalactiae*									
10^3^ CFU/mL	79	71	2	90	45	0	85	0	0
10^4^ CFU/mL	81	79	50	93	50	0	100	0	0
*E. coli*									
10^3^ CFU/mL	88	84	84	98	81	0	91	76	3
10^4^ CFU/mL	92	93	99	87	64	0	89	61	1

^a^: Time post-contact. ^b^ Standard error of the survival rate is in the range of 0–0.05% for all the values.

**Table 6:** Comparison of median survival rates of the three major mastitis pathogens in response to the additivated S5 and S6 elastomers and control calculated using the Kaplan–Meier method (differences between PAA-added and control elastomers were all significant at *p* = 0.001).

Pathogen	Median Survival Rate (%)								
	Control			S5			S6		
*S. aureus*	T1^a^	T6	T24	T1	T6	T24	T1	T6	T24
10^3^ CFU/mL	98 ^b^	79	6	90	57	1	96	67	2
10^4^ CFU/mL	86	77	8	93	68	1	95	67	4
*S. agalactiae*									
10^3^ CFU/mL	79	71	2	100	0	0	100	51	0
10^4^ CFU/mL	81	79	50	100	51	0	100	58	0
*E. coli*									
10^3^ CFU/mL	88	84	84	85	74	2	86	68	4
10^4^ CFU/mL	92	93	99	76	50	1	80	61	1

^a^ Time post-contact. ^b^ Standard error of the survival rate is in the range of 0–0.05% for all the values.

**Table 7:** Comparison of median survival rates of the three major mastitis pathogens in response to the additivated R1 elastomer and control calculated using the Kaplan–Meier method (differences between PAA-added and control elastomers were all significant at *p* = 0.001).

Pathogen	Median Survival Rate (%)											
	Control			S7			S8			S9		
*S. aureus*	T1 ^a^		T6	T24	T1		T6		T24	T1		T6
10^3^ CFU/mL	98 ^b^	79	6	99	75	7	95	76	26	98	77	5
10^4^ CFU/mL	86	77	8	95	86	26	96	62	17	93	87	18
*S. agalactiae*												
10^3^ CFU/mL	79	71	2	91	81	6	98	81	14	94	86	22
10^4^ CFU/mL	81	79	50	99	84	0.4	99	72	6	97	84	8
*E. coli*												
10^3^ CFU/mL	88	84	84	97	93	27	99	71	7	94	78	22
10^4^ CFU/mL	92	93	99	96	93	15	100	98	31	99	95	8

^a^ Time post-contact. ^b^ Standard error of the survival rate is in the range of 0–0.05% for all the values.

## Data Availability

Data are not available due to presence of patent applications.
